# Methotrexate elicits pro-respiratory and anti-growth effects by promoting AMPK signaling

**DOI:** 10.1038/s41598-020-64460-z

**Published:** 2020-05-12

**Authors:** David J. Papadopoli, Eric H. Ma, Dominic Roy, Mariana Russo, Gaëlle Bridon, Daina Avizonis, Russell G. Jones, Julie St-Pierre

**Affiliations:** 1 Department of Biochemistry, McGill University, Montréal, QC H3G 1Y6 Canada; 2 Goodman Cancer Research Centre, Montréal, QC H3A 1A3 Canada; 3 Department of Physiology, McGill University, Montréal, QC H3G 1Y6 Canada; 4 Center for Cancer and Cell Biology, Program in Metabolic and Nutritional Programming, Van Andel Research Institute, Grand Rapids, MI 49503 USA; 5 Department of Biochemistry, Microbiology and Immunology, University of Ottawa, Ottawa, ON K1H 8M5 Canada; 6 Ottawa Institute of Systems Biology, University of Ottawa, Ottawa, ON K1H 8M5 Canada

**Keywords:** Cancer metabolism, Molecular medicine

## Abstract

One-carbon metabolism fuels the high demand of cancer cells for nucleotides and other building blocks needed for increased proliferation. Although inhibitors of this pathway are widely used to treat many cancers, their global impact on anabolic and catabolic processes remains unclear. Using a combination of real-time bioenergetics assays and metabolomics approaches, we investigated the global effects of methotrexate on cellular metabolism. We show that methotrexate treatment increases the intracellular concentration of the metabolite AICAR, resulting in AMPK activation. Methotrexate-induced AMPK activation leads to decreased one-carbon metabolism gene expression and cellular proliferation as well as increased global bioenergetic capacity. The anti-proliferative and pro-respiratory effects of methotrexate are AMPK-dependent, as cells with reduced AMPK activity are less affected by methotrexate treatment. Conversely, the combination of methotrexate with the AMPK activator, phenformin, potentiates its anti-proliferative activity in cancer cells. These data highlight a reciprocal effect of methotrexate on anabolic and catabolic processes and implicate AMPK activation as a metabolic determinant of methotrexate response.

## Introduction

Cell growth and proliferation require the production of numerous macromolecules. One-carbon metabolism covers a complex metabolic network important for the production of nucleotides, lipids, reducing power, and substrates for methylation reactions^1,2^. Indeed, many genetic and functional studies have highlighted the importance of this pathway, along with hyperactivation of serine/glycine biosynthesis, in driving tumourigenesis^3,4^. Enzymes of the mitochondrial folate pathway, such as methylenetetrahydrofolate dehydrogenase/cyclohydrolase 2 (MTHFD2), which are normally low or absent in normal adult tissues^5,6^, are highly upregulated in cancer and are negatively correlated with survival in breast cancer patients^7^. Also, enzymes implicated in serine biosynthesis, such as phosphoglycerate dehydrogenase (PHGDH), are upregulated in triple-negative breast cancer and melanoma^8,9^, while serine hydroxymethyltransferase 2 (SHMT2) expression and glycine consumption correlate with cancer cell proliferation and poor prognosis across several cancer cell types^10–12^. In support of this point, a recent report showed that patients with colorectal and lung adenocarcinomas that have high expression of mitochondrial one-carbon metabolism genes have shorter overall survival compared to those with low expression of these genes^13^.

The effectiveness of targeting one-carbon metabolism in cancer was initially recognized over 60 years ago. In 1948, Sydney Farber discovered that treatment of patients with a folic acid antagonist, aminopterin, produced temporary remission in children with acute lymphoblastic leukemia (ALL)^14^. This landmark paper led the way for the development of a class of drugs called anti-folates. Methotrexate (MTX), a member of this class of drugs, is one of the most commonly used anti-folates in chemotherapy treatment for various cancers, including acute lymphoblastic leukemia, breast cancer, bladder cancer and lymphomas^15^. Although MTX can be used as a single agent, such as to treat choriocarcinoma^16^, it is most commonly used in combination with cyclophosphamide and 5-fluorouracil as part of the CMF (cyclophosphamide, methotrexate, 5-fluorouracil) treatment for breast cancer^17^. MTX inhibits dihydrofolate reductase (DHFR), an enzyme required to produce tetrahydrofolate (THF) that is needed to build nucleotides^18^. MTX also inhibits 5-aminoimidazole-carboxyamide ribonucleotide formyltransferase (ATIC) and thymidylate synthetase (TYMS), which are enzymes of the purine and pyrimidine biosynthesis pathways respectively^19^. Despite its wide use as a cancer drug, MTX can have high toxicity by targeting metabolic enzymes found in both transformed and non-transformed cells, hence reducing its therapeutic index^20,21^. In this manuscript, we uncover that MTX promotes AMPK signaling in breast cancer, which results in stimulation of mitochondrial metabolism and inhibition of cellular proliferation. This knowledge will assist in refining cancer-specific therapeutic strategies involving MTX.

## Results

### Methotrexate induces AMPK signaling

**Figure 1:**
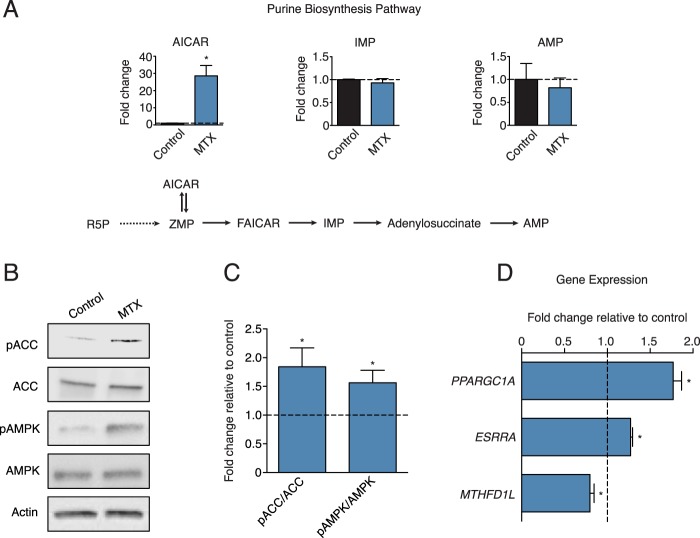
Methotrexate activates AMPK signaling by increasing endogenous AICAR levels. (**A**) Analysis of purine metabolites (AICAR, IMP, AMP) following treatment with 0.1 µM MTX (blue) or control (black) for 72 hours in BT-474 cells, normalized to control treatment (dashed line) (n = 3). (**B**) Immunoblots of phosphorylated-ACC (Ser79), total ACC, phosphorylated-AMPKα (T172), total AMPK, or Actin in BT-474 cells treated with 0.1 µM MTX or control for 72 hours (n = 3). (**C**) Quantitation of immunoblots from (B) (n = 3). (**D**) Expression of *PPARGC1A*, *ESRRA* and *MTHFD1L* in BT-474 cells treated with 0.1 µM MTX (blue) or control for 72 hours, normalized to control treatment (dashed line) (n = 3). Full length blots are presented in Supplementary Fig. 3. All data are presented as means + SEM, *p < 0.05, Student’s *t* test.

Given that MTX inhibits purine biosynthesis, we first quantified the levels of metabolic intermediates in this pathway upon treatment. MTX caused a strong increase (~30 fold) in endogenous AICAR levels in breast cancer cells and mouse embryonic fibroblasts (MEFs) (Fig. 1A, Supplementary Fig. 1), with little effect on the downstream metabolites IMP and AMP, suggesting a blockage in *de novo* purine biosynthesis at the ATIC step. AICAR is used as an exogenous compound to activate AMPK in various cell models^22^, hence we assessed whether the increase in endogenous AICAR levels upon methotrexate treatment was sufficient to promote AMPK activation. MTX treatment increased the phosphorylation of Ser^79^ on acetyl-CoA carboxylase (pACC)^23^, and the phosphorylation of Thr^172^ on AMPK, indicating that AMPK is activated (Fig. 1B,C). PGC-1α signaling is a known downstream effector of AMPK activation in both non-transformed and transformed cells^24–26^. Accordingly, MTX treatment increased the expression of *PPARGC1A* and its partner *ESRRA* in BT-474 cells, indicating that MTX upregulates the PGC-1α/ERRα axis (Fig. 1D). In addition, MTX decreases the expression of *MTHFD1L* (Fig. 1D), a folate cycle gene that is repressed by AMPK/PGC-1α/ERRα signaling^26^. Collectively, these data show that MTX treatment promotes AMPK signaling.

### Methotrexate promotes AMPK-dependent mitochondrial respiration

**Figure 2:**
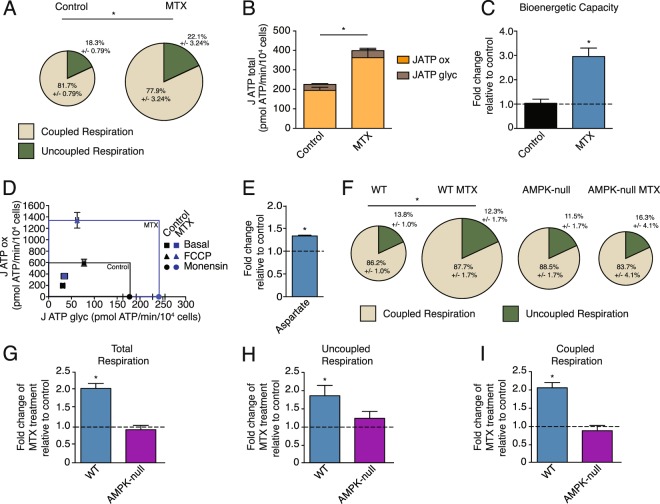
Methotrexate promotes cellular respiration and increases global bioenergetic capacity in an AMPK-dependent manner. (**A**) Respiration of BT-474 cells treated with 0.1 µM MTX or control for 72 hours. Size of pie chart indicates fold change of total respiration upon MTX treatment (Fold change of 1.98 of MTX-treated cells compared to control); % of coupled respiration (beige) and uncoupled respiration (green) are shown (n = 4). (**B**) Quantification of total ATP production (J ATP total) for BT-474 cells treated with 0.1 µM MTX or control for 72 hours under basal conditions (10 mM glucose). J ATP total is the sum of J ATP ox (oxidative phosphorylation, orange) and J ATP glyc (glycolysis, brown) (n = 3). (**C**) Quantification of total bioenergetic capacity in BT-474 cells treated with 0.1 µM MTX (blue) or control (black), compared to control treatment (dashed line) (n = 3). (**D**) Bioenergetic capacity of BT-474 cells treated with 0.1 µM MTX (blue box/data points) or control (black box/data points). Data points represent J ATP values as a function of J ATP glyc (x-axis) and J ATP ox (y-axis) after the following additions: basal (square), FCCP (triangle), monensin (circle). The size of the box represents the theoretical bioenergetic space that can be occupied by each condition (n = 3). (**E**) Aspartate levels of BT-474 cells treated with 0.1 μM MTX (blue) for 72 hours, normalized to control treatment (dashed line) (n = 3). (**F**) Respiration of WT and AMPK-null MEFs treated with 0.02 µM MTX or control for 72 hours. Size of pie chart indicates fold change of total respiration upon MTX treatment (Fold change of WT MTX: 2.13, AMPK-null: 1.25, AMPK-null MTX: 0.93, compared to WT); % of coupled respiration (beige) and uncoupled respiration (green) are shown (n = 4). (**G–I**) Total, uncoupled, and coupled respiration of WT MEF cells treated with MTX (blue) and AMPK-null MEF cells treated with MTX (purple) compared to each respective control (dashed line) (n = 4). All data are presented as means + SEM, *p < 0.05, Student’s *t* test for (**A–C,E,G–I**), and two-way ANOVA, Dunnett’s post hoc test for (**F**).

To test the biological implications of AMPK activation upon MTX treatment, we first performed respirometry experiments given that AMPK engages the PGC-1α/ERR axis, which is a central regulator of mitochondrial oxidative phosphorylation. In accordance with the role of AMPK in promoting catabolic reactions, MTX increased cellular respiration in breast cancer cells and non-transformed mammary cells, including the respiration linked to ATP synthesis (coupled respiration) and the respiration linked to proton leak (uncoupled respiration) (Fig. 2A, Supplementary Fig. 2A–F). We also formally quantified the impact of MTX on global cellular bioenergetics^28^. MTX treatment increased basal total cellular ATP production (J ATP total), which was largely due to an increase in oxidative phosphorylation (J ATP ox), with a small contribution from glycolysis (J ATP glyc) (Fig. 2B). MTX treatment also increased maximal total bioenergetic capacity (Fig. 2C,D) and the levels of aspartate, a metabolite linked to increased respiration in proliferating cells^27^ (Fig. 2E). In addition, MTX promoted mitochondrial metabolism in non-transformed MEFs. Indeed, MEFs treated with MTX displayed increased total, uncoupled and coupled respiration at baseline, similar to cancer cells (Fig. 2F,G–I blue bars). To determine if the MTX-induced increase in oxidative metabolism was AMPK-dependent, MEF cells deficient for AMPKα1/2 were treated with MTX. AMPK-null MEF cells showed no significant increase in oxidative metabolism upon MTX treatment (Fig. 2F,G–I purple bars). Taken together, these results demonstrate that MTX promotes mitochondrial respiration in an AMPK-dependent manner.

### Methotrexate exerts AMPK-dependent anti-proliferative effects

**Figure 3:**
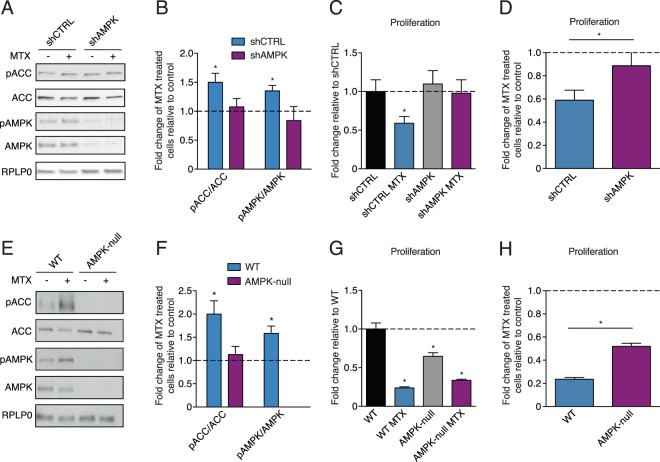
Methotrexate decreases cellular proliferation in an AMPK-dependent manner. (**A**) Immunoblots of phosphorylated-ACC (Ser79), total ACC, phosphorylated-AMPKα (T172), total AMPK, and RPLP0 in eμ-Myc B cell lymphoma cells transfected with shCTRL or shAMPK and treated with 2 nM MTX or control for 72 hours (n = 3). (**B**) Quantitation of immunoblots from (A) (n = 3). (**C**) Cell proliferation of eμ-Myc B cell lymphoma cells transfected with shCTRL or shAMPK treated with 2 nM MTX for 72 hours compared to shCTRL control (dashed line) (n = 4). (**D**) Cell proliferation of eμ-Myc B cell lymphoma cells transfected with shCTRL or shAMPK and treated with MTX compared to each respective control (dashed line) (n = 4). (**E**) Immunoblots of phosphorylated-ACC (Ser79), total ACC, phosphorylated-AMPKα (T172), total AMPK, and RPLP0 in WT and AMPK-null MEF cells treated with 0.05 μM MTX or control for 72 hours (n = 3). (**F**) Quantitation of immunoblots from (**E**) (n = 3). (**G**) Cell proliferation of WT and AMPK-null MEF cells treated with 0.05 μM MTX for 72 hours compared to WT control (dashed line) (n = 3). (H) Cell proliferation of WT and AMPK-null MEF cells treated with MTX compared to each respective control (dashed line) (n = 3). Full length blots are presented in Supplementary Figs. 4 and 5. All data are presented as means + SEM, *p < 0.05, Student’s *t* test for (**B,D,F,H**), and two-way ANOVA, Dunnett’s post hoc test for (**C,G**).

It is well established that MTX acts as a chemotherapeutic agent by inhibiting cellular proliferation^29^. Given that AMPK-mediated metabolic reprogramming has previously been shown to impact proliferation and tumour growth^30,31^, we tested if the anti-proliferative effect of MTX is dependent on AMPK. We first examined this in eμ-Myc B cell lymphoma cells using an AMPKα1/α2 hairpin construct (shAMPK) targeting AMPK. Knockdown of AMPK in cancer cells decreased the anti-proliferative effects of MTX compared to controls (shCTRL) (Fig. 3A–D). Similarly, the proliferation of AMPK-null MEF cells was less inhibited by MTX treatment than WT controls (Fig. 3E–H). Together, these results indicate that the growth inhibitory effects of MTX are dependent on AMPK.

### Formate supplementation does not rescue the AMPK-dependent effects of MTX treatment

**Figure 4:**
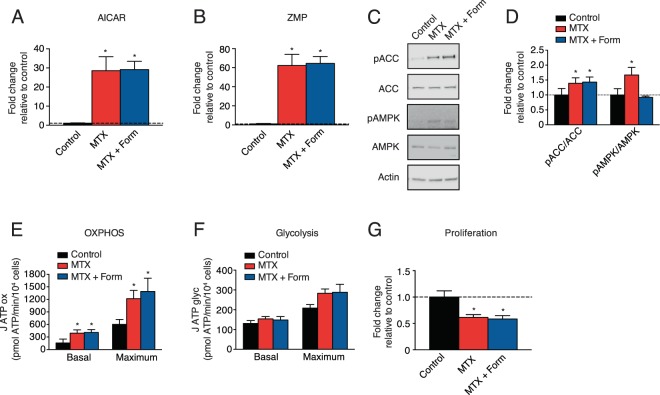
Formate does not rescue the AMPK-dependent metabolic effects of MTX. (**A,B**) Analysis of metabolites (AICAR and ZMP) following treatment with 0.1 µM MTX (red) or a combination of 0.1 µM MTX and 1 mM sodium formate (dark blue) in BT-474 cells for 72 hours, normalized to control treatment (n = 3). (**C**) Immunoblots of phosphorylated-ACC (Ser79), total ACC, phosphorylated-AMPKα (T172), total AMPK, or Actin in BT-474 cells treated with MTX, MTX and formate, or control for 72 hours (n = 3). (**D**) Quantitation of immunoblots from (**C**) (n = 3). (E-F) Quantification of basal and maximal oxidative ATP production (J ATP ox) and glycolytic ATP production (J ATP glyc) in BT-474 cells treated with MTX, MTX and formate, or control for 72 hours (n = 3). (G) Cell proliferation of BT-474 cells treated with MTX, MTX and formate, or control for 72 hours, compared to control (dashed line) (n = 5). Full length blots are presented in Supplementary Fig. 6. All data are presented as means + SEM, *p < 0.05, one-way ANOVA, Dunnett’s post hoc test (**A,B,D-G**).

Genetic defects in one-carbon metabolism have been rescued by formate supplementation^32^. To determine if the AMPK-dependent effects of MTX treatment can be rescued by formate supplementation, BT-474 cells were treated with a combination of MTX and sodium formate. Formate supplementation did not rescue the MTX-mediated induction of AICAR or ZMP, which resulted in unchanged AMPK signaling activation (Fig. 4A–D), and unaltered cellular bioenergetics (Fig. 4E,F). Furthermore, formate did not rescue the MTX-mediated decrease in cell count (Fig. 4G). Ultimately, the AMPK-dependent effects of MTX do not appear to arise due to lack of formate.

### Biguanides potentiate the anti-neoplastic effect of methotrexate

**Figure 5:**
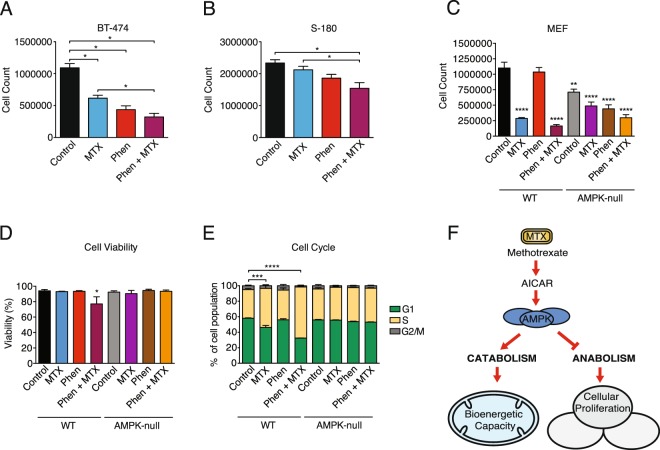
Phenformin treatment potentiates MTX response. (**A**) Cell proliferation of BT-474 cells treated with 0.02 mM phenformin or vehicle for 24 hours, then treated with 0.02 mM phenformin or vehicle and 0.1 µM MTX for 72 hours (n = 5). (**B**) Cell proliferation of sarcoma-180 (S-180) cells treated with 0.01 mM phenformin for 24 hours, then treated with 0.01 mM phenformin and 0.01 µM MTX for 72 hours (n = 7) (**C**) Cell proliferation, (**D**) cell viability, and (**E)** cell cycle analysis of WT and AMPK-null MEF cells treated with MTX (0.02 μM), phenformin (0.1 mM) or both for 72 hours (n = 3). (**F**) Schematic indicating that AMPK signaling plays a functional role in controlling the catabolic and anabolic procresses induced by methotrexate. All data are presented as means + SEM, *p < 0.05, ***p < 0.001, ****p < 0.0001, one-way ANOVA, Dunnett’s post hoc test for (**A,B**), and two-way ANOVA, Dunnett’s post hoc test for (**C–E**).

Cancer cells are dependent on one-carbon metabolism to build nucleotides *de novo*^2^. Recently, we have shown that one-carbon metabolism is inhibited through activation of AMPK/PGC-1α/ERRα signaling, resulting in increased sensitivity to MTX^26^. In addition, the AMPK/PGC-1α/ERRα axis is activated by treatment with biguanides^24,26,33–35^. To investigate whether co-treatment with biguanides can potentiate the anti-proliferative effects of MTX, cells were pre-treated with phenformin for 24 hours before treatment with MTX for 72 hours. Cells treated with both phenformin and methotrexate demonstrated lower cell counts than those treated with methotrexate or phenformin alone (Fig. 5A,B). AMPK-null MEF cells were also less sensitive to the combination of phenformin and MTX in terms of cell proliferation, viability, and cell cycle progression compared to WT cells (Fig. 5C–E), further highlighting the impact of AMPK on MTX response. These data show that activation of AMPK using phenformin can potentiate the anti-proliferative effect of MTX in cancer cells.

## Discussion

In this study, we report that AMPK signaling plays a key role in mediating the metabolic and anti-proliferative effects of MTX (Fig. 5F). MTX treatment increases endogenous levels of AICAR, leading to AMPK activation. Increased AMPK signaling results in a decrease in one-carbon metabolism gene expression and cell proliferation. In addition, elevated AMPK signaling augments cellular global bioenergetic capacity, mainly by promoting oxidative phosphorylation. The anti-proliferative effects of MTX was potentiated by phenformin, a complex I inhibitor that activates AMPK, which suppresses one-carbon metabolism through PGC-1α/ERRα signaling^26^. These data highlight potential synergy between MTX and modulators of mitochondrial bioenergetics, and underscore the importance of understanding the metabolic impact of cancer drugs to improve their chemotherapeutic action.

MTX is a potent inhibitor of one-carbon metabolism and nucleotide biosynthesis. Previous work has shown that MTX treatment causes an accumulation of AICAR^36^ sufficient to activate AMPK in breast cancer cells^37–40^. MTX-induced AMPK signaling was shown to promote glucose uptake and lipid oxidation^39^, as well as inhibit protein synthesis^40^. The anti-folate drug pemetrexed also induces AICAR-mediated AMPK activation and inhibits anabolic processes in colon cancer cells and lymphoblastic leukemia cells^41,42^. Here, we show that MTX does not only inhibit anabolic processes, but also promotes catabolic metabolism by stimulating mitochondrial respiration.

Our group has previously shown that the AMPK/PGC-1α/ERRα axis can inhibit one-carbon metabolism and purine biosynthesis, resulting in decreased proliferation^26^. Given that the anti-proliferative effects of MTX are linked to AMPK activity, we show in this study that the AMPK activator phenformin can improve the anti-proliferative action of MTX. This is line with reports showing that pharmacological AMPK activation with AICAR also increases the antineoplastic effect of MTX in breast, skin, and prostate cancer models^37^. However, given that AICAR has poor bioavailability^43^, combining MTX with AMPK activators that have higher bioavailability, such as biguanides, may prove to be an effective option in the clinic.

There has been increased interest in repurposing biguanides for cancer treatment. Indeed, both phenformin and metformin display AMPK-dependent and –independent anti-neoplastic effects in several cancer subtypes including breast, prostate, and colorectal cancers^44,45^. Despite these successes, clinical trials regarding the usage of biguanides in cancer have been disappointing^44^. Given that biguanides and MTX both share a common effector in AMPK, the combination of both drugs may have strong clinical implications in improving AMPK-dependent anti-neoplastic response. Indeed, AMPK has been reported to have both anti-Warburg and anti-proliferative effects^31^. However, the overall outcome of AMPK activation in cancer is still unclear as AMPK can limit anabolic processes required for proliferation, yet promote metabolic adaptation of tumours to cope under energetic stress^46^.

Overall, our results highlight the importance of AMPK in mediating chemosensitivity. Our work will likely stimulate translational research aimed at testing whether biguanides could be used as neoadjuvant therapy to improve chemotherapeutic response to anti-folates.

## Methods

### Cell lines and reagents

BT-474, S-180, MCF10A, MCF7, and NMUMG cell lines were purchased from ATCC and cultured as previously described^26,47^. NT2196 is a stable cell line corresponding to immortalized NMuMG cells that were transformed with an oncogenic form of Neu (ErbB2), which was cultured as previously described^48^. AMPK-null MEFs and eμ-Myc B cell lymphoma shCTRL/shAMPK cell lines were generous gifts from Dr. Russell Jones’ laboratory, and were cultured as previously described^31,45,49^. The eμ-Myc B cell lymphoma cell line was engineered using the plasmid MSCV p2GM AMPK alpha2hp1 alpha1hp1 (Addgene plasmid # 89492; http://n2t.net/addgene:89492; RRID:Addgene_89492). Methotrexate (MTX) (dissolved in DMSO) and phenformin (dissolved in water) was purchased from Sigma. All LC/MS grade solvents and salts were purchased from Fisher (Ottawa, Ontario Canada: water, acetonitrile (ACN), Methanol (MeOH), formic acid, and ammonium acetate. The authentic metabolite standards were purchase from Sigma-Aldrich Co. (Oakville, Ontario, Canada).

### Western Blotting

Protein extracts were prepared using lysis buffer (50 mmol/L Tris-HCl [pH 7.4], 1% Triton X-100, 0.25% sodium deoxycholate, 150 mmol/L NaCl, 1 mmol/L EDTA) supplemented with protease inhibitors (cOmplete Mini EDTA-free Protease Inhibitor Cocktail, cat# 4693159001) and phosphatase inhibitors (PhosphoStop cat# 4906837001). Immunoblots were incubated with the following primary antibodies from Cell Signaling: pAMPK (2531), AMPK (2532), pACC (3661), ACC (3662); from Santa Cruz: Actin (sc-1616); RPLP0 (11290-AP) The results were visualized with Western Lightning Plus-ECL (PerkinElmer) and analyzed with ImageJ software (NIH).

### Quantitative RT-PCR

RNA was extracted and purified using the Aurum Total RNA Mini Kit (Bio-Rad) following the manufacturers’ protocols. Reverse transcriptase reactions were performed using iScript cDNA Synthesis Kit (Bio-Rad). Samples were then analyzed by qRT-PCR with SYBR-green-based qRT-PCR with a MyiQ2 Real-Time Detection System (Bio-Rad). Gene-specific primers are found in Supplementary Table 1. Values were normalized to *PUM1*.

### Respirometry

Cellular respiration was determined using a Digital Model 10 Clark Electrode (Rank Brothers) as previously described^50^. 1 × 10^6^ cells (BT-474, MCF10A, MCF7) or 1.5 × 10^6^ (NMuMG, NT2196) cells were treated with methotrexate for 72 hours and used for respiration measurements. Oligomycin (2.5 μg/mL/1 × 10^6^ cells) was added to inhibit the ATP synthase, which allows for the calculation of respiration coupled to ATP synthesis (coupled respiration) and respiration linked to proton leak (uncoupled respiration). Myxothiazol (0.5 μM/1 × 10^6^ cells) was added to inhibit complex III, and is used to determine the contribution of non-mitochondrial respiration, which was not observed.

### Seahorse bioenergetic analysis

Oxygen Consumption Rate (OCR) and Extracellular Acidification Rate (ECAR) were measured using the XFe24 Extracellular Flux Analyzer (Agilent Technologies, Santa Clara, CA, USA) according to the manufacturer’s protocol. BT-474 cells were treated for 72 hours with methotrexate, then trypisizinzed and 50,000 cells per well were plated overnight in Seahorse cell culture plates (Agilent Technologies, Santa Clara, CA, USA). The next day, cells were washed with XF media (Seahorse Bioscience) without glucose and incubated in a CO_2_-free incubator at 37 °C for 2 hours to establish equilibrium. Basal conditions include XF media with 10 mM glucose. Oligomycin (1 μM), FCCP (1.5 μM), rotenone and antimycin A (0.5 μM each), and monensin (20 μM) were used. Oligomycin is an inhibitor of ATP synthase. FCCP uncouples the inner mitochondrial membrane, thereby allowing for maximal oxygen consumption. The combination of rotenone (complex I inhibitor) and antimycin A (complex III inhibitor) is used to maximally perturb mitochondrial respiration. Monensin is used to calculate the maximum glycolytic capacity of cells, by driving increased ATP demand by the Na^+^/K^+^–ATPase. In addition, HCl (hydrochloric acid) was injected into wells containing media with no cells (four injections of 0.25 mM each) in order to calculate the buffer capacity of the XF media. OCR, ECAR, and PPR (proton production rate) measurements were taken before and after each injection, and were used to calculate ATP production (J ATP total) and bioenergetic capacity as previously described^28^. Briefly, bioenergetic capacity is the product of the maximal ATP from glycolysis (J ATP glyc) and ATP from oxidative phosphorylation (J ATP ox). Values were normalized to cell counts.

### Cell proliferation

Cell counts were quantified using an automated TC10 cell counter (Bio-Rad) and viability was determined using trypan blue exclusion.

### Metabolomics

Nucleotide detection and analysis was performed using LC-MS/MS at the Metabolomics Core Facility of the Goodman Cancer Research Centre. Cultured cells were treated with methotrexate for 72 hours. Cells were washed in ammonium formate three times, then quenched in cold 50% methanol (v/v) and acetonitrile. Cells were lysed following bead beating at 30 Hz for 2 minutes. Cellular extracts were partitioned into aqueous and organic layers following dichloromethane treatment and centrifugation. Aqueous supernatants were dried down using a refrigerated speed-vac. Dried samples were subsequently resuspended in 25 μl of water. A 10 μl volume of sample was injected onto an Agilent 6430 Triple Quadrupole (QQQ)-LC-MS/MS for targeted metabolite analysis of AICAR (5-aminoimidazole-4-carboxamide-1-β-D-ribofuranoside) and a 10-fold dilution was injected for nucleotides: ZMP (5-aminoimidazole-4-carboxamide ribonucleotide), IMP (inosine monophosphate), and AMP (adenosine monophosphate). The liquid chromatography separation was performed using a 1290 Infinity ultra-performance binary LC system (Agilent Technologies, Santa Clara, CA, USA). The chromatography run was conducted as follows: column temperature was maintained at 10 °C and the separation was realised by reverse phase separation using a flow rate of 0.4 mL/min with a Scherzo SM-C18 column 3 μm, 3.0 × 150 mm (Imtakt Corp, JAPAN) The gradient started at 100% mobile phase A (5 mM ammonium acetate in water) with a 5 min gradient to 100% B (200 mM ammonium acetate in 80% water/20% ACN) at a flow rate of 0.4 ml/min. This was followed by a 5 min hold time at 100% mobile phase B and a subsequent re-equilibration time (6 min) before next injection.

The mass spectrometer was equipped with an electrospray ionization (ESI) source and samples were analyzed in positive ionization mode. Multiple reaction monitoring (MRM) transitions were optimized on standards for each metabolite quantitated. Transitions for quantifier and qualifier ions were as follows: AICAR (259.1 → 127.0 and 110.0, 82.1, 55.1), ZMP (339.1 → 127.0 and 110.0), IMP (349.0 → 147 and 110.0), and AMP (348.0 → 146.1 and 348.0 → 118.9). Gas temperature and flow were set at 350 °C and 10 l/min respectively, nebulizer pressure was set at 40 psi and capillary voltage was set at 3500 V. Relative concentrations were determined from external calibration curves prepared in water and compared to sample area under the curve. Note that no corrections were made for ion suppression or enhancement. Data were analyzed using MassHunter Quant (Agilent Technologies, Santa Clara, CA, USA).

### Flow cytometry analysis of cell cycle

WT and AMPK-null MEF cells were seeded in 12-well plates and grown for 24 h, after which they were treated with control, MTX, phenformin or both for 72 h. Cells were counted and 100,000 cells were washed with cold wash buffer (PBS + 5% FBS + 0.01 M NaN_3_), spun down, and resuspended in hypotonic buffer (0.1% sodium citrate, 0.1% Triton X-100 in water). Cells were stained with Propidium iodide (50 μg/ml) (cat #: P4170, Sigma) for 30 min at 37 °C in the dark. Samples were analyzed with a FACS Canto II (San Jose, CA). Fluorescence was detected by excitation at 488 nm and acquisition on the 585/42 PI-A channel.

## Supplementary information

Supplementary information.

## Data Availability

The datasets generated and/or analysed during the current study are available from the corresponding author on reasonable request.
