# Supplementary material for: Methotrexate elicits pro-respiratory and anti-growth effects by promoting AMPK signaling
Source: Sci Rep. 2020 May 12;10:7838. doi: 10.1038/s41598-020-64460-z (PMC7217946; doi:10.1038/s41598-020-64460-z)
Supplement: Supplementary file 1 — Supplementary information. [file 41598_2020_64460_MOESM1_ESM.docx]

**Methotrexate elicits pro-respiratory and anti-growth effects by promoting AMPK signaling**

David J. Papadopoli, Eric Ma, Dominic Roy, Mariana Russo, Gaëlle Bridon, Daina Avizonis, Russell G. Jones, and Julie St-Pierre

**Supplementary Figure 1. Methotrexate increases endogenous AICAR levels.** Analysis of AICAR levels in MEF cells following treatment with 0.02μM MTX or control for 72 hours. Data are normalized to control treatment (dashed line) (n=3). Data are presented as means + SEM, *p<0.05, Student’s *t* test.

**Supplementary Figure 2. Methotrexate increases cellular respiration.** (A-C) Total, uncoupled, and coupled respiration of MCF10A and MCF7 cells treated with MTX compared with control treatment (dashed line) (n=9-10). (D-F) Total, uncoupled, and coupled respiration of NMuMG and NT2196 cells treated with MTX compared with control treatment (dashed line) (n=4). Data are presented as means + SEM, *p<0.05, Student’s *t* test.

**Supplementary Figure 3.** Original western blots for Figure 1B.

**Supplementary Figure 4.** Original western blots for Figure 3A.

**Supplementary Figure 5.** Original western blots for Figure 3E.

**Supplementary Figure 6.** Original western blots for Figure 4C.

**Supplementary Table 1: Primers for qRT-PCR**
